# The Preference for Internet-Based Psychological Interventions by Individuals Without Past or Current Use of Mental Health Treatment Delivered Online: A Survey Study With Mixed-Methods Analysis

**DOI:** 10.2196/mental.5324

**Published:** 2016-06-14

**Authors:** Emma Emmett Karolina Wallin, Susanne Mattsson, Erik Martin Gustaf Olsson

**Affiliations:** ^1^Department of PsychologyUniversity of UppsalaUppsalaSweden; ^2^Department of Public Health and Caring SciencesUniversity of UppsalaUppsalaSweden

**Keywords:** patient acceptance of health care, patient preference, patient satisfaction, Internet-based cognitive behavioral therapy, chronic disease, mental health care, eHealth, implementation, qualitative research

## Abstract

**Background:**

The use of the Internet has the potential to increase access to evidence-based mental health services for a far-reaching population at a low cost. However, low take-up rates in routine care indicate that barriers for implementing Internet-based interventions have not yet been fully identified.

**Objective:**

The aim of this study was to evaluate the preference for Internet-based psychological interventions as compared to treatment delivered face to face among individuals without past or current use of mental health treatment delivered online. A further aim was to investigate predictors of treatment preference and to complement the quantitative analyses with qualitative data about the perceived advantages and disadvantages of Internet-based interventions.

**Methods:**

Two convenience samples were used. Sample 1 was recruited in an occupational setting (n=231) and Sample 2 consisted of individuals previously treated for cancer (n=208). Data were collected using a paper-and-pencil survey and analyzed using mixed methods.

**Results:**

The preference for Internet-based psychological interventions was low in both Sample 1 (6.5%) and Sample 2 (2.6%). Most participants preferred psychological interventions delivered face to face. Use of the Internet to search for and read health-related information was a significant predictor of treatment preference in both Sample 1 (odds ratio [OR] 2.82, 95% CI 1.18-6.75) and Sample 2 (OR 3.52, 95% CI 1.33-9.29). Being born outside of Sweden was a significant predictor of preference for Internet-based interventions, but only in Sample 2 (OR 6.24, 95% CI 1.29-30.16). Similar advantages and disadvantages were mentioned in both samples. Perceived advantages of Internet-based interventions included flexibility regarding time and location, low effort, accessibility, anonymity, credibility, user empowerment, and improved communication between therapist and client. Perceived disadvantages included anonymity, low credibility, impoverished communication between therapist and client, fear of negative side effects, requirements of computer literacy, and concerns about confidentiality.

**Conclusions:**

Internet-based interventions were reported as the preferred choice by a minority of participants. The results suggest that Internet-based interventions have specific advantages that may facilitate help-seeking among some individuals and some disadvantages that may restrict its use. Initiatives to increase treatment acceptability may benefit from addressing the advantages and disadvantages reported in this study.

## Introduction

Increasing the access to evidence-based mental health services is crucial for improving global health [1]. The use of information technology, such as computers, mobile phones, and tablets—referred to as eHealth—is a relatively new way to promote self-care and well-being in a health care setting [2]. The use of the Internet has the potential to increase access to proven mental health services for a far-reaching population at a low cost [3]. For example, Internet-based cognitive behavioral therapy (iCBT) shows promising results for the treatment of common mental health problems, such as depression and anxiety disorders [4,5], and for improving psychosocial outcomes among individuals coping with somatic conditions such as chronic pain [4]. Internet-based interventions may be especially beneficial for individuals with somatic health conditions, due to its flexibility with regard to service use [6].

Although promising, implementation of Internet-based interventions in routine care has proven to be challenging [7]. This study builds on previous studies that investigate acceptability as a key determinant for successful implementation of Internet-based interventions. Research into treatment acceptability originates from the idea that a given intervention needs to be both effective and acceptable for intended users. A treatment is acceptable when it is perceived as appropriate, fair, reasonable, and nonintrusive for a given problem [8]. Treatment acceptability is important to consider since it may improve both adherence [9] and overall outcome [10]. For example, according to a meta-analytic review across different treatment formats and target populations, individuals that had been matched to a preferred treatment had a 58% chance of showing improvements and were nearly half as likely to drop out of treatment compared to those that did not receive their preferred choice of treatment [10].

Internet-based interventions have several advantages over traditional face-to-face delivery, some of which may improve treatment acceptability. Internet-based interventions typically consist of text-based material, which may enhance treatment fidelity and save therapist time [5]. Interventions may be used either as a sole treatment component or as a complement to other forms of treatment [11]. Some interventions include support from a therapist, for example, through mail or telephone. In a qualitative review, reasons for providing care via the Internet included reduction of health service costs, increased convenience for users, overcoming isolation of users, increased user and health provider control of the intervention, and stigma reduction [12]. Among primary care patients participating in an iCBT intervention for depression, major advantages include flexibility with regard to time and location of service use, and making it easier to fit therapy into daily life [13]. Internet-based interventions may also provide a sense of anonymity that encourages shy and embarrassed users to be more open about themselves [14].

Treatment acceptability among patients in Internet-based interventions is generally high. In a meta-analysis of iCBT for mood disorders, the results indicated adequate adherence, with a median of 80% of the included participants completing all steps. Patient satisfaction was also high, with a median of 86% of the participants reporting that they were satisfied or very satisfied [5]. Similarly, in a review about iCBT for individuals with clinical levels of depression in routine care, positive expectancies and high satisfaction were reported [15]. While dropout rates were comparable to other formats of treatment, take-up rates were lower, ranging from 3% to 25%. However, in many studies a more detailed evaluation of treatment acceptability or satisfaction was lacking. The authors also raised concerns about low take-up rates, and concluded that the low take-up rates may either indicate a reluctance to take part in research or a reluctance to enter Internet-based interventions [15].

Treatment acceptability has been framed as a key factor for successful implementation in routine care. A given treatment may be clinically effective, yet unacceptable for patients [15]. A number of factors have been identified among individuals with past or current experience of Internet-based interventions related to treatment acceptability. In a qualitative study, some patients reported feeling more comfortable writing about their thoughts and feelings at their own pace, while others expressed a concern about how to develop a relationship with a virtual therapist and to communicate emotional content via a computer [13]. Moreover, patients commonly report concerns about privacy, confidentiality, and the trustworthiness of the system [16]. It has also been suggested that inadequate Internet provision and low levels of education are associated with a decreased likelihood of using Internet-based interventions [17].

Efforts to investigate treatment acceptability among individuals without past or current use of mental health treatment delivered online have been made. In a study recruiting primary care patients with an interest in some sort of behavioral treatment, approximately half (48%) considered the Internet as a valid treatment format, while the majority (92%) preferred face-to-face care [18]. When investigating predictors, time constraints were related to a higher interest in Internet-based interventions while symptom severity was not. In another study, individuals from the general population were recruited to fill out an online survey about the perceived acceptability of Internet-based interventions [19]. The sample consisted mainly of female university students that used the Internet daily. The results indicated a lower likelihood to use Internet-based interventions compared to face-to-face interventions. No significant differences were observed with regard to factors such as gender, previous or current mental health status, or computer literacy. Furthermore, Internet-based interventions only met participants’ expectations in terms of convenience of access. Dissatisfaction was expressed regarding important factors for engaging in treatment, such as perceived helpfulness, the ability to motivate users, credibility, appeal, and feedback.

In sum, Internet-based interventions will have limited impact if potential users do not perceive them as acceptable. Previous studies have investigated the acceptability of Internet-based interventions among both individuals with and without past or current experience of them. However, these studies often employ online samples or patients already taking part in treatment programs. The representativeness of the participants included can therefore be questioned [20,21]. For example, patients with ongoing interventions may already have overcome some practical and stigma-related barriers for seeking psychological help. Therefore, the aim of this study was to investigate the acceptability of Internet-based interventions among individuals without past or current experience of mental health treatment delivered online. More specifically, we wanted to investigate the preference for Internet-based psychological interventions as compared to treatment delivered face to face. Furthermore, predictors of treatment preference were investigated. As a complement to the quantitative analysis, qualitative data about the perceived advantages and disadvantages were analyzed.

This study included two samples: one sample consisted of individuals recruited from a general occupational setting; another sample consisted of individuals previously treated for cancer. The latter sample was selected to represent a potential target group, since Internet-based interventions may have several positive effects for people coping with chronic diseases such as cancer [22,23]. Furthermore, the use of a paper-and-pencil survey was chosen to reach individuals less familiar with the use of computers and the Internet. This study may provide knowledge about the generalizability of previous studies, as well as new insights into potential barriers and facilitators for implementation of Internet-based interventions in routine care.

## Methods

### Participants

Two convenience samples were recruited. One sample was recruited in an occupational setting (Sample 1). The second sample was recruited in cancer clinics and consisted of individuals previously treated for cancer (Sample 2). To be included, participants had to be over 18 years old, and able to read and write Swedish. Individuals with prior or current experience of mental health treatment delivered online, and individuals that had not used the Internet during the past two years, were excluded.

In Sample 1, 243 individuals working in a university setting (67/243, 27.6%) and a rural factory (176/243, 72.4%) were recruited. Of these 243 participants, 12 (4.9%) were excluded because they had not used the Internet during the past two years (5/243, 2.1%), had prior experience of psychological treatment via the Internet (3/243, 1.2%), or failed to answer questions on prior Internet usage (4/243, 1.6%). Of the remaining 231 out of the initial 243 participants (95.1%), 74 (32.0%) identified themselves as women and 157 (68.0%) as men. The mean age of Sample 1 was 44.1 years (SD 12.2).

In Sample 2, 285 individuals were recruited from outpatient, postcancer follow-up clinics. Only participants that had completed cancer treatment were included. Some participants were excluded because they had not used the Internet or computers during the past two years (70/285, 24.6%), or had prior experience of psychological treatment via the Internet (7/285, 2.5%). Of the remaining 208 out of the initial 285 participants (73.0%), 91 (43.8%) identified themselves as women and 117 (56.3%) as men. The mean age of Sample 2 was 60.5 years (SD 13.9). Among women, most had been treated for breast cancer (34/165, 20.6%), followed by lymphoma (18/165, 10.9%). Time since first diagnosis among women ranged from 1 to 33 years (mean 6.5 years). Among men, the most common diagnosis for previous treatment was prostate cancer (61/274, 22.3%), followed by lymphoma (10/274, 3.6%). Time since first diagnosis among men ranged from 2 to 33 years (mean 6.8 years). Further demographic description and analysis of differences between the two samples are presented in Table 1.

**Table 1:** Demographic description and analysis of differences between samples.

Variable		Sample 1, n (%) or mean (SD)	Sample 2, n (%) or mean (SD)	χ^2^ or *t*	*P*
Total, n (%)		231 (100)	208 (100)		
**Gender, n (%)**					
	Women	74 (32.0)	91 (43.8)	χ^2^_1_=6.40	.01
	Men	157 (68.0)	117 (56.3)		
Age in years, mean (SD)		44.1 (12.2)	60.5 (13.9)	*t*_429_=13.10	<.001
**Civil status, n (%)**					
	In a relationship	169 (73.2)	176 (84.6)	χ^2^_1_=9.88	.002
	Single	62 (26.8)	30 (14.4)		
**Education, n (%)**					
	High school	127 (55.0)	103 (49.5)	χ^2^_1_=1.41	.24
	College/university	100 (43.3)	102 (49.0)		
**Country of birth, n (%)**					
	Born in Sweden	211 (91.3)	185 (88.9)	χ^2^_1_=0.49	.49
	Born outside of Sweden	20 (8.7)	22 (10.6)		
HOSQ^a^*reading* factor, mean (SD)		8.96 (6.43)	9.96 (9.17)	*t*_332.34_=-1.27	.21
HOSQ *interacting* factor, mean (SD)		2.73 (3.75)	4.92 (6.91)	*t*_278.24_=-3.89	<.001

^a^HOSQ: Health Online Support Questionnaire.

As shown in Table 1, participants in Sample 1 were older, more often female, and more often in a relationship as compared to those in Sample 2. There were no significant differences between the two samples in terms of level of education, country of birth, or the use of interacting online support. Participants in Sample 2 used the Internet for interactional support significantly more than the participants in Sample 1.

### Procedure

Data were collected using a paper-and-pencil survey. Sample 1 was recruited using an advertisement posted at the workplace for 2 months. The survey contained written information about the study. Approximately 500 surveys were handed out. In total, 243 surveys out of 500 (48.6%) were completed and returned to the researcher by post.

Sample 2 was recruited from a university hospital. Recruitment lasted for 4 months. Eligible participants received verbal and written information about the study in the waiting room of the clinics—oncology and urology. Included participants filled out the survey in conjunction with their visit or at home, and returned the survey to the researchers by post. In total, 350 surveys were handed out and 285 (81.4%) were completed. The participants received no financial compensation.

### Materials

#### Demographics

Data including age, gender, civil status (single/in a relationship), level of education (high school/university), country of birth (born in Sweden/born outside of Sweden), diagnosis, and year since diagnosis (only participants previously treated for cancer) were collected using a customized questionnaire.

**Health-Related Online Support**

The Health Online Support Questionnaire (HOSQ) is a self-report that measures the use of online support for health problems [24]. The HOSQ consists of 18 items rated on a 6-point Likert scale, ranging from 0 (not relevant/never) to 5 (on a daily basis). The scale is divided into two subscales— *reading* and *interacting—* with an equal number of items. *Reading* refers to searching for and reading health-related information online to improve health or to make informed decisions about treatments. *Interacting* refers to sharing health-related information, seeking encouragement, or communicating with others regarding health-related issues online. The two HOSQ subscales have shown adequate internal consistency (Cronbach alpha=.88 and .77, respectively) and content and construct validity [24]. In this study, the two HOSQ subscales showed adequate internal consistency in both Sample 1 (*reading* Cronbach alpha=.88; *interacting* Cronbach alpha=.76) and Sample 2 (*reading* Cronbach alpha=.92; *interacting* Cronbach alpha=.86).

#### Treatment Preference

Participants were asked to indicate which treatment modality they would prefer if in need of psychological help now or in the future: face to face, Internet, or both modalities to an equal extent. Internet-based psychological treatment was described as a program delivered via the Internet with or without support from an online therapist. Participants could also indicate that they would never seek any psychological treatment.

#### Perceived Advantages and Disadvantages of Internet-Based Interventions

By means of open-ended questions, participants were invited to list perceived advantages and disadvantages of psychological treatment via the Internet separately on three blank lines each.

### Statistical Analysis

A total of 96 out of 439 (21.9%) participants—77 in the nonclinical and 19 in the clinical sample—did not respond to the question regarding treatment preference, and were therefore not included in further analyses. The variables HOSQ *reading* and HOSQ *interacting* showed a positively skewed distribution. A median split was conducted prior to analysis on both variables to create binary variables (high and low). Although a median split reduces the variability of continuous variables, it is a way to enhance clarity when group differences are in focus [25]. Multivariate logistic regressions were conducted to investigate predictors of treatment preference. A forced-entry approach was used since no hypothetical relationship was assumed between the predictors. Due to a low preference for Internet-based interventions in both samples, the criterion variable was collapsed into two levels: (1) preference for face-to-face intervention (used as reference category) and (2) preference for Internet or equal preference for both modalities. SPSS version 20 (IBM Corp) was used for the statistical analyses.

### Qualitative Analysis

Qualitative content analysis was used to analyze the open-ended questions regarding perceived advantages and disadvantages of Internet-based interventions. Qualitative content analysis is a method to systematically condense and organize data into categories describing a phenomenon of interest [26]. In this study, the open-ended answers were considered the unit of analysis. Advantages and disadvantages were treated as different content areas and analyzed separately. As a first step, the answers were divided into meaning units by one of the authors (EEKW). A meaning unit was considered as a part of data that conveyed enough information to provide a sense of meaning. Next, the same author (EEKW) condensed the meaning units by taking away redundant wording without changing the meaning or core content.

An inductive approach was applied to Sample 1, as is recommended when knowledge is missing or fragmented [26]. In an inductive approach, meaning units are arranged into categories with different levels of abstraction according to similarities and differences to create mutually exclusive categories [26,27]. To improve the credibility, two independent coders (the authors EMGO and SM) used a deductive approach to verify the initial categorization. Codes placed in different or more than one category were discussed and revised by the coders (EEKW, EMGO, and SM) to create mutually exclusive categories.

In Sample 2, a deductive approach was used to test the replicability of the categorization generated from Sample 1. Three coders (EEKW, EMGO, and SM) conducted this process independently. Only minor changes were made to the categorization obtained from Sample 1. As a final step, categories with similar content were grouped and labeled with an overarching theme (ie, categorizing the categories).

### Ethical Approval

The study was approved by the Regional Ethical Review Board in Uppsala, Sweden (2013-11-20; Diary number 2013/436).

## Results

### Preference for Internet-Based Interventions

The preference for Internet-based psychological interventions was low in both samples. In Sample 1, the results showed that out of the 154 participants that responded to the question regarding treatment preference, 103 (66.9%) preferred face-to-face treatment, 10 (6.5%) preferred treatment provided via the Internet, and 32 (20.8%) preferred both formats of delivery to an equal extent. A total of 9 (5.8%) participants indicated that they would not prefer any treatment modality if needed now or in the future.

Similar results were obtained with Sample 2. There was no significant difference when comparing treatment preference between the two samples (χ^2^_2_=2.7, *P*=.26). The results showed that in Sample 2, out of the 189 participants that responded to the question regarding treatment preference, 123 (65.1%) preferred face-to-face treatment, 5 (2.6%) preferred treatment provided via the Internet, and 41 (21.7%) preferred both modalities to an equal extent. A total of 20 (10.6%) participants indicated that they would not prefer any treatment modality if needed now or in the future.

Only participants that indicated a preference for face-to-face, Internet, or both modalities equally were included in further analyses. The results showed a significant difference between treatment preferences in both Sample 1 (χ^2^_2_=97.8, *P*<.001) and in Sample 2 (χ^2^_2_=129.9, *P*<.001). Post hoc analyses in Sample 1 showed that a significantly higher number of participants preferred psychological treatment provided face to face compared to via the Internet (χ^2^_1_=76.5, *P*<.001) and both equally (χ^2^_1_=37.3, *P*<.001). A significantly higher number of participants also preferred both modalities equally compared to the Internet as first choice (χ^2^_1_=11.5, *P*=.001).

In Sample 2, post hoc analyses showed that a significantly higher number of participants preferred psychological treatment delivered face to face compared to via the Internet (χ^2^_1_=108.8, *P*<.001) and both equally (χ^2^_1_=41, *P*<.001). A significantly higher number of participants also preferred both modalities equally compared to the Internet as first choice (χ^2^_1_=28.2, *P*<.001). These results suggest that most people preferred face-to-face psychological treatment, followed by both modalities equally. Internet-based interventions were the least preferred format of treatment.

### Predictors of Treatment Preference

Multivariate logistic regressions were used to analyze predictors of treatment preference. The variables age, sex, civil status, education, country of birth, HOSQ *reading*, and HOSQ *interacting* were included in the analyses using a forced-entry approach. The results showed that a test of the full model had a low but acceptable goodness of fit in Sample 1 (Cox and Snell R^2^=.08; Nagelkerke R^2^=.11). Overall prediction success was 71.6%. Only HOSQ *reading* made a significant unique contribution in the prediction of treatment preference (*P*=.02). The odds ratio (OR) indicates that participants who already use online health-related information are 2.82 times more likely than those who do not to report a preference for Internet or both Internet and face-to-face treatment equally if in need of psychological help now or in the future (see Table 2).

**Table 2:** Summary of logistic regression for variables predicting treatment preference in Sample 1.

Variables	OR^a^	95% CI	*P*
Age	1.00	0.97-1.04	.93
Sex (reference: woman)	1.05	0.45-2.47	.91
Civil status (reference: in a relationship)	1.42	0.60-3.35	.43
Education (reference: low)	1.36	0.60-3.11	.46
Country of birth (reference: born in Sweden)	0.70	0.16-3.10	.64
HOSQ^b^*reading* (reference: low)	2.82	1.18-6.75	.02
HOSQ *interacting* (reference: low)	1.33	0.56-3.17	.52

^a^OR: odds ratio.

^b^HOSQ: Health Online Support Questionnaire.

In Sample 2, the result from the multivariate logistic regression showed that a test of the full model had a low but acceptable goodness of fit (Cox and Snell R^2^=.13; Nagelkerke R^2^=.19). Overall prediction success was 73.3%. HOSQ *reading* made a significant unique contribution in the prediction of treatment preference (*P*=.01). The odds ratio indicates that participants that already use online health-related information were 3.5 times more likely to report a preference for Internet or both Internet and face-to-face treatment equally if in need of psychological help now or in the future. Furthermore, country of birth made a significant contribution in the prediction of treatment preference (*P*=.02). The odds ratio indicates that participants born outside Sweden were 6.2 times more likely to report a preference for Internet or both Internet and face-to-face treatment equally (see Table 3).

Taken together, results from the multivariate logistic regressions suggest that past online behavior, such as searching for and reading health-related information to improve health or make informed decisions about treatments, was positively related to a preference for Internet-based interventions in both samples. Country of birth was a significant predictor only in the sample consisting of individuals previously treated for cancer, suggesting that individuals born outside Sweden are more likely to prefer Internet-based interventions compared to individuals born in Sweden.

### Perceived Advantages and Disadvantages

A total of 116 out of 148 (78.4%) participants in Sample 1 and 113 out of 173 (65.3%) in Sample 2 reported at least one perceived advantage and/or disadvantage related to Internet-based interventions. Sample 1 generated 117 codes related to advantages and 72 codes related to disadvantages. Sample 2 generated 146 codes related to advantages and 134 codes related to disadvantages.

The result of the qualitative content analysis of advantages included the following themes: flexibility regarding time and location, accessibility, low effort, anonymity, credibility, user empowerment, and improved communication. A number of participants reported that Internet-based interventions have no advantages or that they did not know. Perceived advantages are presented as themes, categories, and example codes in Table 4.

With regard to disadvantages, the following themes were obtained from the categorization of codes: anonymity, low credibility, impoverished communication, negative side effects, and computer literacy/safety concerns. Some participants reported that Internet interventions had no disadvantages, or that they did not know. Perceived disadvantages are presented as themes, categories, and example codes in Table 5.

**Table 3:** Summary of logistic regression for variables predicting treatment preference in Sample 2.

Variables	OR^a^	95% CI	*P*
Age	1.01	0.98-1.04	.55
Sex (reference: woman)	1.10	0.45-2.65	.84
Civil status (reference: in a relationship)	0.60	0.17-2.11	.42
Education (reference: low)	1.80	0.77-4.19	.18
Country of birth (reference: born in Sweden)	6.24	1.29-30.16	.02
HOSQ^b^*reading* (reference: low)	3.52	1.33-9.29	.01
HOSQ *interacting* (reference: low)	1.15	0.44-3.02	.77

^a^OR: odds ratio.

^b^HOSQ: Health Online Support Questionnaire.

**Table 4:** Themes, categories, and example codes of perceived advantages of Internet-based interventions.

Themes	Frequency, n (%)		Categories	Example codes
	Sample 1 (n=117)	Sample 2 (n=146)		
**Flexibility regarding time and location**				
	13 (11.1)	17 (11.6)	No transportation	No need to leave home when feeling sick No transportation More comfortable at home No need to go out and meet people No need to visit hospital/treatment facility
	8 (6.8)	10 (6.8)	Independent of time and place	I can work with the program when and where it best suits me Easier to fit into my daily schedule More flexible
	4 (3.4)	11 (7.5)	No need to schedule appointments	No need to schedule/keep appointments Independent of visiting hours
**Low effort**				
	13 (11.1)	9 (6.2)	Time-saving	Quick Less time-consuming Time-effective
	16 (13.7)	5 (3.4)	Cheap	Cost-effective Cheaper for the individual and society Affordable
	11 (9.4)	4 (2.7)	Convenient	Easy Low effort
**Accessibility**				
	8 (6.8)	16 (11.0)	Reach	Accessible for more people Increased access for people working odd hours Increased access for people in rural areas/living abroad Easy to access
	3 (2.6)	12 (8.2)	Always available	Internet available 24/7 Always available
	5 (4.3)	4 (2.7)	No delay	No waiting list/queue Decreased delay of treatment onset Quick treatment onset
**Anonymity**				
	4 (3.4)	7 (4.8)	Integrity	More integrity More anonymous/private
	5 (4.3)	1 (0.7)	Lack of face-to-face contact with a therapist	No need to see a therapist Less embarrassing than seeing a therapist No eye contact—no shame
	5 (4.3)	0 (0)	Nobody needs to know	My family does not need to know More people would dare to seek treatment if anonymous
**Credibility**				
	6 (5.1)	5 (3.4)	Treatment expectancy	Good If I had problems I would use it Interesting
	2 (1.7)	5 (3.4)	Useful information	When you need concrete advice such as checklists Information is saved
	0 (0)	4 (2.7)	Standardized	Treatment less influenced by the individual therapist Independent of the therapist
**User empowerment**				
	2 (1.7)	8 (5.5)	Decide duration	I can initiate/terminate treatment when I want/need Sense of self-help
	1 (0.9)	4 (2.7)	Own pace	Time to reflect Work at your own pace
	0 (0)	2 (1.4)	Choose content	You can choose treatment More alternatives
**Improved communication**				
	7 (6.0)	4 (2.7)	Self-disclosure	Easier to express oneself Easier to be open and honest Easier to write than to tell face to face Dare to ask
None	1 (0.9)	15 (10.3)	None	N/A^a^
Do not know	3 (2.6)	3 (2.1)	Do not know	N/A
Total	117 (100)	146 (100)	N/A	N/A

^a^N/A: not applicable.

**Table 5:** Themes, categories, and example codes of perceived disadvantages of Internet-based interventions.

Themes	Frequency, n (%)		Categories	Example codes
	Sample 1 (n=72)	Sample 2 (n=134)		
**Anonymity**				
	6 (8)	64 (47.8)	Lack of face-to-face contact with a therapist	Human contact irreplaceable/helpful in itself Impersonal Dehumanizing
	8 (11)	10 (7.5)	Lack of empathy and trust	Lack of compassion/trust/affirmation Less closeness in the conversation Less empathic
**Low credibility**				
	12 (17)	9 (6.7)	Unable to motivate	Would not take it seriously Risk of not going through with the treatment Demands self-discipline
	9 (13)	6 (4.5)	Less effective	Impossible to involve the family Less effective Unprofessional
	3 (4)	2 (1.5)	Standardized	Less personal Less individualized
	1 (1)	0 (0)	Incorrect information	Incorrect/excessive information
**Impoverished communication**				
	5 (7)	3 (2.2)	Absence of body language	Lack of eye contact Not being able to read facial expressions and reactions of the therapist
	3 (4)	5 (3.7)	Lack of instant feedback	Inadequate feedback Noninteractive Lack of follow-up questions
	2 (3)	3 (2.2)	Misunderstandings	Risk of misunderstandings Difficult to understand the treatment
	1 (1)	2 (1.5)	Difficulties expressing oneself	Difficult to express myself in writing Not being able to make myself understood
**Negative side effects**				
	3 (4)	9 (6.7)	Self-isolation	I will be alone and isolated at home Nobody to talk to
	5 (7)	6 (4.5)	Risk of incorrect decisions	Risk of wrong diagnosis Difficult to identify symptoms and people in need of more help
**Computer literacy/ safety concerns**				
	4 (6)	2 (1.5)	Confidentiality concerns	Integrity problems Do not trust computers
	4 (6)	1 (0.7)	Require technique and technical skills	Demands computer skills and willingness to use a computer Demands access to Internet
None	4 (6)	5 (3.7)	None	N/A^a^
Do not know	2 (3)	7 (5.2)	Do not know	Do not know how it works
Total	72 (100)	134 (100)	N/A	N/A

^a^N/A: not applicable.

The relative percentage of codes in each theme differed between the samples in some aspects. Low effort was reported more often as an advantage in Sample 1 (34%) compared to Sample 2 (12%). Anonymity was reported more frequently as a disadvantage in Sample 2 (55%) compared to Sample 1 (19%). Moreover, treatment credibility was reported more often as a disadvantage in Sample 1 (35%) compared to Sample 2 (13%). In sum, the result of the qualitative content analysis indicates similar themes in both samples. Some of the themes were presented both as an advantage and as a disadvantage, for example, anonymity and aspects related to credibility and the quality of communication.

## Discussion

### Principal Findings

The aim of this study was to investigate the preference for Internet-based psychological interventions in individuals without past or current experience of them. In line with previous studies, we found that the preference for Internet-based interventions was low [18,28]. Most participants preferred psychological treatment delivered face to face. Only 6.5% and 2.6% in Samples 1 and 2, respectively, reported a clear preference for Internet-based interventions. Although a higher percentage of participants reported that they thought the Internet would be an equally preferable alternative to face-to face treatment, the results of this study suggest an overall low acceptability of Internet-based interventions in both samples.

Participants that often use the Internet to find information in order to improve health or make informed decisions about treatments were approximately three times more likely to prefer an Internet-based psychological intervention across samples. This result suggests that those who already use online health-related support hold more positive attitudes toward Internet-based psychological treatment. It is also possible that HOSQ *reading* represents general computer literacy, Internet familiarity, and everyday Internet use not only restricted to health issues. On the other hand, sharing health-related information, seeking encouragement, or communicating with others regarding health-related issues online was not related to a preference for Internet-based interventions. However, the mean use of interacting support was low in both samples, which makes these findings less reliable.

In contrast to previous findings, level of education was unrelated to treatment preference [17]. Instead, country of birth was an unexpected significant predictor among participants previously treated for cancer. Individuals born outside of Sweden were about six times more likely to prefer an Internet-based intervention compared to those born in Sweden. In a systematic review, it was found that ethnic minorities are disproportionately deterred by stigma when seeking mental health services [29]. As reflected in the qualitative content analysis, the anonymity of Internet interventions may provide a less stigmatizing alternative to formal mental health services that may explain a higher preference for treatment delivered via the Internet.

The qualitative part of this study generated similar advantages and disadvantages as have previously been reported [12-14,16]. It appears that individuals without past experience of Internet-based interventions have a relatively clear idea about factors that they perceive as potential facilitators and barriers. Moreover, although similar themes were present in both samples, the relative frequency of perceived advantages and disadvantages varied. For example, low effort was reported more frequently as an advantage among the participants in the working population. This might be explained by the fact that individuals previously treated for cancer were older and therefore more likely to be retired and to have fewer time constraints. Moreover, anonymity appeared to be of greater concern among individuals previously treated for cancer. This may reflect more experience with health care as a result of past medical treatment for cancer. It might also be explained by the fact that the participants in this sample were older, on average. In a study about cognitive behavioral therapy (CBT) for depression provided face to face, older patients reported nonverbal communication and talking to a therapist as beneficial more often than younger patients [30].

### Limitations

This study has several limitations. First, the response rate in Sample 1 was relatively low (49%). Furthermore, a large number of the participants did not respond to the questions regarding treatment preference. It is possible that this reflects a systematic bias, in which individuals with negative attitudes toward psychological treatment failed to respond to the survey.

Secondly, narrow or incomplete answers to the open-ended questions sometimes gave rise to uncertainty of the intended meaning in the qualitative content analysis, which may influence both the credibility and transferability of the categorization of advantages and disadvantages. The use of in-depth interviews may have generated a more thorough understanding of the specific categories. Furthermore, the nature of the qualitative content analysis does not permit analysis of the relative importance of different categories in the actual decision to seek care when needed.

Finally, the preference related to treatment modality was measured by the use of a single item. Using more items would likely have generated a more reliable overall score. It is also important to note that the question about treatment preference should be considered hypothetical since it is likely that many of the included participants do not perceive a current need for psychological treatment.

### Implications

The results of this study may prove useful to understand more about the acceptability of Internet-based interventions in a number of ways. A relatively high number of individuals previously treated for cancer were excluded because they had not used the Internet during the past two years. Although the Internet may no longer be considered a new technology, low computer literacy and poor access to the Internet have previously been reported as barriers for engagement in Internet-based interventions [17]. In our study, perceived disadvantages related to the treatment format, such as a fear of negative side effects, computer literacy and safety concerns, low credibility, and fear of impoverished communication indicates a general distrust and reluctance to engage in an Internet-based intervention. Concerns about negative side effects and safety have also been reported among individuals that have completed Internet-based interventions [31]. Although Internet-based interventions are commonly secured by encrypting and double authentication, concerns about confidentiality and safety may limit its attractiveness [16]. Hence, in order to improve the uptake of Internet-based interventions, researchers and care providers may want to consider ways to address these barriers.

Internet-based interventions were perceived as empowering for the user in a number of ways. Participants reported that they believe the Internet format gives the user more control over the content, duration, initiation, and termination of treatment. Some individuals wrote that they would feel more comfortable writing about their thoughts and feelings. In addition to this, individuals also reported that Internet-based interventions may provide a sense of anonymity, which may help them overcome fear or discomfort associated with going or talking to a health care professional. A recent study suggested that mixing online and face-to-face treatment provides an opportunity to obtain optimal benefits from the advantages of both treatments [32]. The authors conclude that in order to enable added benefits, individual abilities, needs, and preferences should be considered in a structured way. Engagement of potential stakeholders in the process of developing interventions may improve both uptake rates and the outcome of eHealth interventions [2].

### Conclusions

This study suggests that the preference for Internet-based interventions is low. The past use of online health-related informational support emerged as a significant predictor across samples. Although Internet-based interventions may have specific perceived advantages compared to treatment delivered face to face, low acceptability appears to be an important barrier for large-scale dissemination. To promote large-scale service utilization, it might be beneficial to address the perceived disadvantages reported in this study. When doing so, practitioners and researchers may consider ways to address issues related to communication, fear of negative side effects, and concerns related to computer literacy and safety.

## Abbreviations

CBT: cognitive behavioral therapy
HOSQ: Health Online Support Questionnaire
iCBT: Internet-based cognitive behavioral therapy
N/A: not applicable
OR: odds ratio
U-CARE: Uppsala University Psychosocial Care
